# Brucellose humaine : trois visages d’une zoonose insidieuse

**DOI:** 10.48327/mtsi.v6i1.2026.821

**Published:** 2026-02-19

**Authors:** Manel ENNACEUR, Khaoula SOULI, Sonia CHOUAIEB

**Affiliations:** 1 Unité de bactériologie, Service des laboratoires, Hôpital Habib Thameur, Tunis, Tunisie; 2 Faculté de pharmacie de Monastir, Tunisie

**Keywords:** Brucellose, Zoonose, Diagnostic, Antibiothérapie, Tunisie, Afrique du Nord, Brucellosis, Zoonosis, Diagnosis, Antibiotic therapy, Tunisia, North Africa

## Abstract

**Introduction:**

La brucellose humaine est une zoonose fréquente en Tunisie, particulièrement dans les zones rurales à forte activité d’élevage. Sa présentation clinique est très polymorphe, pouvant se manifester sous des formes localisées ou systémiques. Nous rapportons ici trois observations illustrant cette diversité clinique.

**Observation 1:**

Un homme de 69 ans, diabétique, vivant en zone rurale, consulte pour une tuméfaction cervicale douloureuse. La tomodensitométrie révèle une cellulite et une pyomyosite cervicales. La culture du pus issu de la cellulite était positive à *Brucella* spp. Le patient a été traité par doxycycline et rifampicine pendant 6 semaines, avec drainage chirurgical, et l’évolution a été favorable.

**Observation 2:**

Un homme de 48 ans, usager de drogues injectables, a été hospitalisé pour fièvre prolongée associée à un souffle cardiaque. L’échocardiographie a révélé la présence d’une végétation tricuspide (34×24 mm) et une insuffisance tricuspide modérée. L’hémoculture a permis d’isoler *Brucella* spp. Le traitement a consisté en une association antibiotique incluant doxycycline et rifampicine, complétée par une chirurgie valvulaire. L’évolution a été favorable sous cette prise en charge combinée.

**Observation 3:**

Un patient cirrhotique de 50 ans a été admis pour encéphalopathie hépatique fébrile, non améliorée par antibiothérapie empirique. L’hémoculture a isolé *Brucella* spp. Le traitement adapté a conduit à une nette amélioration clinique.

**Conclusion:**

La brucellose humaine présente des manifestations cliniques variées qui compliquent son diagnostic. En Tunisie, la vigilance clinique est essentielle pour un diagnostic précoce et une prise en charge adaptée, en particulier chez les patients à risque. Des études prospectives sont nécessaires pour mieux définir les protocoles thérapeutiques, surtout face aux formes complexes.

## Abstract (en)

**Introduction:**

Human brucellosis is a prevalent zoonotic disease in Tunisia, especially in rural areas with significant livestock activity. The disease can manifest as localized or systemic forms, and its clinical presentation is highly polymorphic. Here, we present three cases that illustrate this clinical diversity.

**Observation 1:**

A 69-year-old diabetic man living in a rural area presented with a painful cervical swelling. Computed tomography revealed cervical cellulitis and pyomyositis. Culture of the pus from the cellulitis yielded positive results for *Brucella* spp., and the patient was treated with doxycycline and rifampicin for six weeks, along with surgical drainage. The outcome was favorable.

**Observation 2:**

A 48-year-old male intravenous drug user was hospitalized due to a prolonged fever associated with a heart murmur. Echocardiography revealed tricuspid vegetation measuring 34×24 mm and moderate tricuspid regurgitation. A blood culture revealed *Brucella* spp., and treatment consisted of a combination of antibiotics, including doxycycline and rifampicin, as well as valve surgery. The outcome was favorable with this combined treatment.

**Observation 3:**

A 50-year-old cirrhotic patient was admitted with febrile hepatic encephalopathy that did not improve with empirical antibiotic therapy. Blood cultures isolated *Brucella* spp., and appropriate treatment led to a marked clinical improvement.

**Conclusion:**

Human brucellosis presents with a variety of clinical manifestations that complicate its diagnosis. In Tunisia, clinical vigilance is essential for the early diagnosis and appropriate management of the disease, particularly in at-risk patients. Prospective studies are needed to better define treatment protocols, especially for complex forms of the disease.

## Introduction

La brucellose humaine, une zoonose causée par des bactéries du genre *Brucella*, est responsable d’un important problème de santé publique dans de nombreux pays [13]. L’incidence humaine mondiale est estimée à plus de 500 000 nouveaux cas chaque année [13]. La transmission aux humains survient principalement par la consommation de produits laitiers non pasteurisés ou par contact direct avec des animaux infectés [1]. Sur le plan clinique, la maladie se caractérise classiquement par une fièvre ondulante associée à des sueurs profuses et des algies diffuses dans les formes aiguës. Les atteintes viscérales focalisées articulaires, neurologiques, génito-urinaires, hépatiques ou encore hématologiques évoquent parfois des tableaux pseudo-tuberculeux [6].

Au-delà de ces manifestations typiques, la brucellose peut également se présenter sous des formes atypiques, subaiguës ou chroniques, rendant le diagnostic plus difficile et pouvant entraîner des retards de prise en charge [6].

En Tunisie, la brucellose demeure endémoépidémique chez l’homme et dans les cheptels, constituant une maladie à déclaration obligatoire. L’incidence des cas humains a nettement augmenté au cours de la dernière décennie, passant de 2,9 pour 100 000 habitants en 2008 à 9,8 en 2017 [4,10], période durant laquelle le nombre de cas a augmenté de 284 en 2005 à 997 en 2018 [7]. Cette hausse pourrait s’expliquer par la persistance de la brucellose animale, la consommation continue de produits laitiers non pasteurisés, ainsi que par l’amélioration des systèmes de surveillance et de notification des cas. Plus de 80 % des cas déclarés proviennent des régions du Sud, notamment Gafsa, Kasserine, Tozeur et Kébili, où l’élevage constitue une activité majeure, faisant de la maladie une affection à prédominance professionnelle. En revanche, dans les régions du Nord-Ouest et du Centre (Béja, Jendouba, Kairouan, Sidi Bouzid), la transmission est principalement liée à la consommation de produits d’origine animale non pasteurisés [10].

Dans ce contexte, la diversité des présentations cliniques, parfois trompeuses, peut conduire à méconnaître des localisations inhabituelles. Nous rapportons ici trois observations illustrant des formes atypiques de brucellose humaine, témoignant de la variabilité clinique de cette infection.

## Observation 1 : abcès cervical à *Brucella*

Un homme de 69 ans résidant en zone rurale à Siliana, diabétique, a été admis le 4 juin 2022 au service de chirurgie ORL pour une masse latérocervicale droite douloureuse, évoluant depuis trois jours. À l’examen clinique, la masse était molle, douloureuse et mesurait 7 cm de grand axe. L’oropharynx était libre et l’otoscopie sans particularités. L’examen neurologique ne retrouvait aucune anomalie. La tomodensitométrie (TDM) cervico-thoracique a objectivé une collection sousmandibulaire droite, spontanément hyperdense, à paroi rehaussée, mesurant 41×38 mm en coupe axiale et 78 mm de hauteur. Cette collection s’étendait vers les espaces parotidien et masticateur. L’ensemble évoquait une cellulite cervicofaciale avec collection trans-spatiale hyperdense, associée à une pyomyosite du muscle sternocléi-do-mastoidien homolatéral.

Les examens biologiques, incluant l’hémogramme, le taux de prothrombine (TP), le bilan rénal, hépatique et l’ionogramme sanguin, étaient normaux à l’exception d’une CRP élevée à 142 mg/l. L’exploration chirurgicale avec dissection progressive a permis le drainage d’un pus franc. Après trois jours d’incubation sur gélose au sang et gélose au sang cuit, de très petites colonies punctiformes non hémolytiques ont été isolées. La coloration de Gram montrait de petits coccobacilles Gram négatif. Les tests catalase et oxydase étaient positifs. L’uréase était positive en 30 minutes, et orientait vers *Brucella* spp. confirmée par le système Vitek^®^2 GN (bioMérieux, Marcy-l’Etoile, France). Lors de la reprise de l’interrogatoire, le patient a rapporté une consommation régulière de lait cru non pasteurisé, probablement à l’origine de la contamination. Un traitement associant doxycycline 200 mg/j et rifampicine 900 mg/j a été instauré pour une durée de 6 semaines avec une bonne évolution.

## Observation 2 : endocardite à *Brucella* chez un usager de drogues injectables

Un homme de 48 ans, ayant des antécédents de psoriasis, de toxicomanie intraveineuse aux opiacés et consommant régulièrement du lait cru non pasteurisé, s’est présenté au service des urgences le 7 septembre 2023 pour une fièvre, des myalgies et des douleurs mictionnelles évoluant depuis trois semaines.

À l’examen clinique, la fréquence respiratoire était de 28 cycles/min, la saturation en oxygène à 94 %, la pression artérielle à 108/59 mmHg et la fréquence cardiaque à 130 battements/min. Pas de souffle ou de bruit surajoutés à l’auscultation cardiaque. L’état de conscience était normal. On notait une hépatomégalie estimée à 20 cm, une température à 38,8 °C et un œdème bilatéral des membres inférieurs. L’électrocardiogramme montrait une tachycardie sinusale. Les analyses biologiques montraient une hyperleucocytose à 11 810/mm³, une anémie hémolytique (Hb : 7 g/dl), une CRP élevée à 361 mg/l ainsi qu’une insuffisance rénale aiguë (urée : 32,8 mmol/l; créatinine : 238 µmol/l). L’examen cytobactériologique des urines était positif, isolant *Enterobacter cloacae*, conduisant initialement au diagnostic d’infection urinaire, rapidement mis hors de cause, la présence d’*E. cloacae* étant considéré comme un contaminant. La TDM a révélé une hépatosplénomégalie avec infarctus splénique, des lésions nodulaires bilatérales des bases pulmonaires évoquant des emboles septiques, ainsi qu’une sacro-iliite. L’échocardiographie réalisée au lit du patient a objectivé une végétation tricuspide (34×24 mm) associée à une insuffisance tricuspide modérée; les cavités cardiaques droites étaient de taille normale. Les hémocultures ont isolé *Brucella* spp. Le traitement antibiotique initial dirigé contre *Staphylococcus aureus* a été remplacé par l’association rifampicine–doxycycline–céfo-taxime. Après six semaines d’antibiothérapie, une échocardiographie de contrôle a montré une réduction de la végétation à 31×21 mm. Le patient a alors bénéficié d’un remplacement valvulaire par bioprothèse. La prise en charge a été compliquée par une bactériémie associée aux soins à *Klebsiella pneumoniae* productrice de carbapénèmase. Par ailleurs, les prélèvements valvulaires peropératoires ont isolé *Brucella* spp. Le patient a poursuivi l’antibiothérapie pendant trois mois. Une évolution favorable a été notée au contrôle à six mois.

## Observation 3 : brucellose systémique révélée par une encéphalopathie hépatique

**Figure 1:**
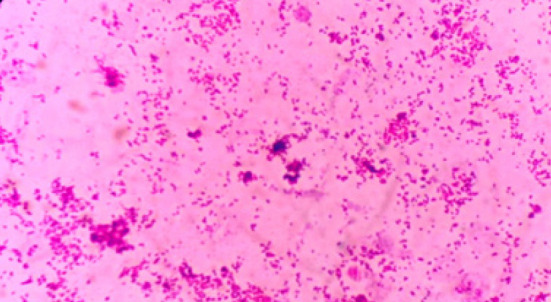
Aspect microscopique de *Brucella* spp. après isolement sur hémoculture

Un patient âgé de 50 ans, ayant comme antécédent principal une cirrhose alcoolique évoluant depuis plusieurs années (stade Child C), a été admis au service des urgences le 20 mars 2024 pour une altération de l’état de conscience. L’examen clinique initial a conclu à une encéphalopathie hépatique de grade III, associée à une fièvre persistante rapportée depuis environ une semaine. Dans ce contexte de cirrhose décompensée fébrile, une antibiothérapie empirique par céfotaxime IV a été initiée. Toutefois, en l’absence d’amélioration et devant la persistance de la fièvre, l’antibiothérapie a été escaladée vers l’imipénème IV. Parallèlement, une enquête infectieuse complète a été menée. L’examen cytobactériologique des urines ainsi que les prélèvements respiratoires sont revenus négatifs, ne permettant pas d’identifier un foyer infectieux évident. Compte tenu du contexte clinique, plusieurs hémocultures ont été réalisées dès l’admission. Au troisième jour d’incubation, l’un des flacons d’hémoculture aérobie s’est positivé. La culture a mis en évidence de petites colonies non hémolytiques. L’examen direct a montré un bacille à Gram négatif, et les tests biochimiques ont révélé des réactions catalase, oxydase et uréase positives, un profil hautement évocateur de *Brucella* spp. (Fig. 1). Face à cette identification, un interrogatoire approfondi a été repris et a mis en évidence une consommation de lait cru, constituant un facteur de risque majeur d’infection à *Brucella.*

L’antibiothérapie a été réajustée selon les recommandations internationales. Une association doxycycline 200 mg/j et rifampicine 900 mg/j a été instaurée pour une durée prévue de 6 semaines. Sous ce traitement, l’évolution a été cliniquement et biologiquement favorable, avec régression progressive du syndrome infectieux et amélioration des paramètres hépatiques et neurologiques. Au terme du bilan, le diagnostic retenu était celui de brucellose systémique survenant chez un patient cirrhotique, responsable de la décompensation hépatique initiale.

## Discussion

Notre série de cas cliniques illustre le polymorphisme clinique de la brucellose.

Le diagnostic à la phase aigüe de la maladie repose sur l’isolement bactérien par culture, bien que lente et peu sensible [8,18]. Dans nos cas, les hémocultures ont permis l’isolement de *Brucella* spp. à partir du 3^è^ jour d’incubation des flacons d’hémoculture. Les caractères phénotypiques observés (bacilles Gram négatif, catalase, oxydase et uréase positives) étaient évocateurs.

Sur le plan thérapeutique, nos trois patients ont été pris en charge conformément aux recommandations internationales, avec une association doxycycline et rifampicine [1].

La première observation illustre une brucellose focale cervico-faciale chez un patient diabétique, un facteur favorisant les formes suppurées. En Tunisie, le diabète est retrouvé avec une fréquence élevée (59 %), bien supérieure à la prévalence moyenne dans d’autres pays, comme l’Algérie (14 % en 2018) [14], sans que cette donnée permette de tirer une conclusion définitive pour notre contexte tunisien. Les études publiées ne rapportent pas de fréquence particulière du diabète au cours de la brucellose [16]. Une prévalence élevée du diabète avait été notée dans les années 1950, mais elle n’a pas été confirmée par des analyses statistiques [12]. Une prédisposition génétique a été suggérée par une étude espagnole en 2003, qui a trouvé une association entre les complications ostéo-articulaires de la brucellose et le gène HLA-B39 [5].

La deuxième observation rapporte un cas d’endocardite brucellienne chez un usager de drogues injectables. L’endocardite infectieuse représente une localisation exceptionnelle de *Brucella*. Elle atteint la valve aortique dans 75 % des cas, puis la valve mitrale, l’association aortique–mitrale et les valves prothétiques, chacune dans 8,3 % des cas [15]. Chez les consommateurs de drogues intraveineuses, la valve tricuspide est habituellement la plus touchée. Les endocardites droites sur valve tricuspide native sont le plus souvent liées aux staphylocoques [17]. Une revue systématique a décrit des endocardites à *Brucella* sur valves prothétiques ou du cœur gauche, mais aucune sur une valve tricuspide native [20]. Dans notre observation, le germe isolé était *Brucella* spp., ce qui confère un caractère exceptionnel à ce cas. La brucellose se manifeste le plus souvent par une fièvre prolongée, des sueurs nocturnes, une asthénie, un malaise général et des arthralgies. Près d’un tiers des patients présente des localisations ostéoarticulaires, qui représentent plus de la moitié des complications [2,9]. Dans notre cas, la sacro-iliite constituait le principal signe évocateur, bien qu’elle ait été initialement confondue avec une infection urinaire. Le diagnostic direct repose sur les hémocultures. Les hémocultures ne sont positives que dans 40 à 70 % des cas selon la littérature [21]. Chez notre patient, l’identification microbiologique a été obtenue sur hémocultures. La présence de végétations tricuspides volumineuses et persistantes (>20 mm) constitue une indication chirurgicale, en raison du risque d’embolie pulmonaire récidivante et d’insuffisance cardiaque droite. Dans notre observation, la végétation mesurait 31×21 mm après six semaines d’antibiothérapie, justifiant l’intervention. Les meilleurs résultats sont obtenus lorsque la chirurgie valvulaire est associée à un traitement antibiotique prolongé [11].

Bien que rare, l’endocardite à *Brucella* est responsable d’environ 80 % de la mortalité liée à la brucellose [19]. Dans notre cas, l’évolution a été favorable après 6 mois de suivi, grâce à la prise en charge combinée médicale et chirurgicale [3]. La 3^è^ observation illustre une brucellose systémique révélée par une décompensation hépatique chez un patient cirrhotique. La cirrhose constitue un état d’immunodépression, exposant à des infections graves et parfois atypiques. Chez ces patients, il est essentiel de considérer non seulement les infections courantes, mais également les infections endémiques locales, telles que la brucellose en Tunisie. La présentation clinique peut être trompeuse, comme dans notre cas, où l’encéphalopathie masquait le caractère infectieux initial. L’interrogatoire ciblé et l’identification de facteurs de risque, notamment la consommation de lait cru, ont été déterminants pour orienter le diagnostic. Cette observation souligne que, chez l’immunodéprimé, la démarche diagnostique doit inclure systématiquement les pathogènes endémiques, afin d’éviter un retard de diagnostic et des complications graves.

Une particularité commune à nos trois observations est le mode de contamination, lié à la consommation de lait cru non pasteurisé. Cette constatation met en évidence l’importance des mesures de prévention à l’interface hommeanimal. Dans le cadre de l’approche *One Health*, qui reconnaît l’interdépendance entre la santé humaine, la santé animale et l’environnement, ces observations soulignent l’importance des mesures de prévention à la fois au niveau humain et animal [1]. Il est donc indispensable d’encourager les services vétérinaires et les services de l’élevage en leur allouant les ressources humaines, financières et logistiques nécessaires, afin d’assurer un meilleur contrôle du volet animal de la maladie, notamment par la surveillance des troupeaux, la vaccination et la pasteurisation des produits laitiers.

Il convient également de rappeler que la transmission peut survenir lors des soins aux animaux ou lors de l’abattage, exposant les individus à des formes cliniques variées, parfois graves, comme les formes systémiques ou ostéo-articulaires [9]. La sensibilisation des populations à ces risques, combinée à des mesures de contrôle vétérinaire strictes, constitue un levier majeur pour réduire l’incidence de la brucellose humaine dans les zones endémiques. Cette stratégie repose notamment sur la promotion de la consommation de produits laitiers pasteurisés, le renforcement de la surveillance sanitaire des troupeaux, ainsi que la mise en œuvre de mesures d’hygiène rigoureuses lors des activités d’élevage et d’abattage.

## Conclusion

La brucellose humaine peut mimer de nombreuses pathologies infectieuses ou inflammatoires, rendant son diagnostic parfois difficile. Dans les zones endémiques comme la Tunisie, elle doit être systématiquement évoquée et recherchée par les praticiens, en particulier chez les patients à risque (vie rurale, consommation de produits laitiers non pasteurisés, professions particulières). La suspicion clinique élevée, associée à une collaboration étroite entre clinicien et microbiologiste, est essentielle pour un diagnostic rapide et une prise en charge efficace.

## Consentement éclairé

Nous avons obtenu le consentement oral des patients.

## Financement

Ce travail n’a bénéficié d’aucun financement.

## Contributions des auteurs et autrices

Manel Ennaceur : conception du rapport de cas, prise en charge diagnostique, rédaction, révision du manuscrit.

Khaoula Souli : prise en charge diagnostique, rédaction.

Sonia Chouaieb : révision et validation du manuscrit.

## Déclaration de liens d’intérêts

Aucun lien d’intérêts lié à ce travail n’a été déclaré.
