# Supplementary material for: Human brucellosis: three faces of an insidious zoonosis
Source: Med Trop Sante Int. 2026 Feb 19;6(1):mtsi.v6i1.2026.821. [Article in French] doi: 10.48327/mtsi.v6i1.2026.821 (PMC13216902; doi:10.48327/mtsi.v6i1.2026.821)
Supplement: Related texts

# Research article: Human brucellosis: three faces of an insidious zoonosis

## Introduction

Human brucellosis, a zoonotic disease caused by bacteria of the genus *Brucella*, is a significant public health problem in many countries [13]. It is estimated that there are more than 500,000 new cases of human brucellosis each year worldwide [13]. Humans can become infected through the consumption of unpasteurized dairy products or direct contact with infected animals [1]. The disease is clinically characterized by undulating fever, profuse sweating, and diffuse pain in acute forms. Visceral involvement of the joints, nervous system, genitourinary system, liver, or hematological system can evoke pseudo-tuberculous presentations [6].

Beyond these typical manifestations, brucellosis can present atypically, subacutely, or chronically forms, which makes diagnosis more difficult and can lead to delays in treatment [6].

In Tunisia, brucellosis is endemic and epidemic in humans and livestock and is a notifiable disease. The incidence of human cases has increased significantly over the last decade, rising from 2.9 to 9.8 per 100,000 inhabitants from 2008 to 2017 [4,10]. During this time, the number of cases increased from 284 in 2005 to 997 in 2018 [7]. This increase could be explained by the persistence of animal brucellosis, the continued consumption of unpasteurized dairy products, and improvements in surveillance and case reporting systems. More than 80% of reported cases come from southern regions, particularly Gafsa, Kasserine, Tozeur, and Kebili, where livestock farming is a major activity. This makes the disease predominantly occupational. In contrast, transmission in the northwestern and central regions (Beja, Jendouba, Kairouan, and Sidi Bouzid) is mainly linked to the consumption of unpasteurized animal products [10].

In this context, the diversity of clinical presentations, which can be misleading at times, can lead to unusual locations being overlooked. Here, we report three observations illustrating atypical forms of human brucellosis, demonstrating the clinical variability of this infection.

## Observation 1: Cervical abscess caused by *Brucella*

A 69-year-old diabetic man living in a rural area of Siliana was admitted to the ENT surgery department on June 4, 2022, for a painful right lateral cervical mass that had developed over the course of three days. Upon examination, the mass was soft and painful with a length of 7 cm. The oropharynx was clear, and otoscopy revealed no abnormalities. Neurological examination revealed no abnormalities. Cervical-thoracic computed tomography (CT) revealed a spontaneously hyperdense submandibular collection on the right side with enhanced walls. The collection measured 41×38 mm in the axial plane and 78 mm in height. This collection extended toward the parotid and masticatory spaces. Overall, the picture was suggestive of cervicofacial cellulitis with a hyperdense trans-spatial collection associated with pyomyositis of the ipsilateral sternocleidomastoid muscle.

Laboratory tests, including a complete blood count, prothrombin time, renal and hepatic function tests, and a blood ionogram, were normal except for an elevated C-reactive protein (CRP) level of 142 mg/L. Surgical exploration with progressive dissection allowed for the drainage of clear pus. After three days of incubation on blood agar and cooked blood agar, very small, nonhemolytic, punctiform colonies were isolated. Gram staining revealed small Gram-negative coccobacilli. Catalase and oxidase tests were positive. The urease test was positive within 30 minutes, indicating *Brucella* spp., which was confirmed by the Vitek^®^2 GN system (bioMérieux, Marcy-l’Etoile, France). Upon further questioning, the patient reported regularly consuming unpasteurized raw milk, likely the source of the contamination. A treatment combining doxycycline (200 mg/day) and rifampicin (900 mg/day) was initiated for six weeks, yielding good results.

## Observation 2: *Brucella* endocarditis in an intravenous drug user

A 48-year-old man with a history of psoriasis, intravenous opioid addiction, and regular consumption of unpasteurized raw milk presented to the emergency department on September 7, 2023, with fever, myalgia, and urinary pain that had been developing for three weeks.

On clinical examination, his respiratory rate was 28 cycles/min, oxygen saturation was 94%, blood pressure was 108/59 mmHg, and heart rate was 130 beats/min. There were no additional sounds or murmurs on cardiac auscultation. His level of consciousness was normal. Hepatomegaly estimated at 20 cm, a temperature of 38.8°C, and bilateral edema of the lower limbs were noted. The electrocardiogram showed sinus tachycardia. Laboratory tests showed hyperleukocytosis at 11,810/mm³, hemolytic anemia (Hb: 7 g/dl), elevated CRP at 361 mg/l, and acute renal failure (urea: 32.8 mmol/l; creatinine: 238 µmol/l). The urinalysis was positive for *Enterobacter cloacae*, which initially led to a diagnosis of a urinary tract infection. However, this diagnosis was quickly ruled out because the presence of *E. cloacae* was considered contamination. CT scan revealed hepatosplenomegaly with splenic infarction, bilateral nodular lesions in the lung bases suggestive of septic emboli, and sacroiliitis. Bedside echocardiography revealed tricuspid vegetation (34×24 mm) associated with moderate tricuspid regurgitation; the right heart chambers were of normal size. Blood cultures isolated *Brucella* spp. The initial antibiotic treatment targeting *Staphylococcus aureus* was replaced by a combination of rifampicin, doxycycline, and cefotaxime. After six weeks of antibiotic therapy, a follow-up echocardiogram showed a reduction in the vegetation to 31×21 mm. The patient then underwent valve replacement with a bioprosthesis. Management was complicated by healthcare-associated bacteremia with carbapenemase-producing *Klebsiella pneumoniae*. In addition, intraoperative valve samples isolated *Brucella* spp. The patient continued antibiotic therapy for three months. A favorable outcome was noted at the six-month follow-up.

## Observation 3: Systemic brucellosis manifesting as hepatic encephalopathy

A 50-year-old patient with a history of alcoholic cirrhosis that progressed over several years (Child stage C) was admitted to the emergency department on March 20, 2024, due to altered consciousness. The initial clinical examination revealed grade III hepatic encephalopathy associated with a persistent fever that had lasted about a week. Given the context of decompensated cirrhosis and fever, empirical antibiotic therapy with intravenous cefotaxime was initiated. However, given the persistence of fever in the absence of improvement, antibiotic therapy was escalated to IV imipenem. At the same time, a complete infectious disease investigation was conducted. Urine cytology and bacteriology tests, as well as respiratory samples, were negative, making it impossible to identify an obvious source of infection. Given the clinical context, several blood cultures were performed upon admission. On the third day of incubation, one of the aerobic blood culture bottles tested positive. The culture revealed small, nonhemolytic colonies. Direct examination revealed a Gram-negative bacillus, and biochemical tests showed positive reactions for catalase, oxidase, and urease, indicating a high likelihood of *Brucella* spp. infection (Fig. 1). After identifying the organism, we conducted a thorough interview, which revealed the patient’s consumption of raw milk, a major risk factor for *Brucella* infection.

**Figure 1:** Microscopic appearance of *Brucella* spp. after isolation on blood culture

Antibiotic therapy was adjusted according to international recommendations. The patient was prescribed a combination of doxycycline (200 mg/day) and rifampicin (900 mg/day) for six weeks. The patient’s clinical and biological condition improved under this treatment, with gradual regression of the infectious syndrome and improvement in hepatic and neurological parameters. The final diagnosis was systemic brucellosis in a cirrhotic patient responsible for the initial hepatic decompensation.

## Discussion

Our series of clinical cases illustrates the clinical polymorphism of brucellosis.

In the acute phase of the disease, diagnosis is based on bacterial isolation by culture, though this method is slow and not very sensitive [8,18]. In our cases, blood cultures allowed for the isolation of *Brucella* spp. from the third day of incubating the blood culture bottles. The observed phenotypic characteristics (Gram-negative bacilli, catalase positive, oxidase positive, and urease positive) were suggestive.

Our three patients were treated in accordance with international recommendations with a combination of doxycycline and rifampicin [1].

The first case presents focal cervicofacial brucellosis in a diabetic patient, a condition that promotes suppurative forms. In Tunisia, diabetes is prevalent at a rate of 59%, which is well above the average prevalence in other countries, such as Algeria (14% in 2018) [14]. However, this data does not allow us to draw a definitive conclusion in the Tunisian context. Published studies do not report a particular frequency of diabetes in brucellosis [16]. A high prevalence of diabetes was noted in the 1950s, but statistical analyses have not confirmed this finding [12]. A Spanish study from 2003 suggested a genetic predisposition, finding an association between osteoarticular complications of brucellosis and the HLA-B39 gene [5].

The second observation reports a case of brucellosis endocarditis in an intravenous drug user. Infectious endocarditis is an unusual location for *Brucella*. It affects the aortic valve in 75% of cases, followed by the mitral valve, the aortic-mitral combination, and prosthetic valves, each in 8.3% of cases [15]. Among intravenous drug users, the tricuspid valve is typically most affected.

Right-sided endocarditis on native tricuspid valves is most often associated with staphylococci [17]. A systematic review described *Brucella* endocarditis on prosthetic valves or the left heart but not on a native tricuspid valve [20]. In our observation, the isolated germ was *Brucella* spp., which makes this case exceptional. Brucellosis typically presents with prolonged fever, night sweats, asthenia, general malaise, and arthralgia. Nearly one-third of patients have osteoarticular involvement, accounting for more than half of complications [2,9]. In our case, sacroiliitis was the main suggestive sign, though it was initially misdiagnosed as a urinary tract infection. Direct diagnosis is based on blood cultures. According to the literature, blood cultures are positive in only 40 to 70% of cases [21]. In our patient, microbiological identification was obtained from blood cultures.

The presence of large, persistent tricuspid vege-tations (>20 mm) indicates the need for surgery due to the risk of recurrent pulmonary embolism and right heart failure. In our observation, the vegetation measured 31×21 mm after six weeks of antibiotic therapy, which justified the intervention. The best results are obtained when valve surgery is combined with prolonged antibiotic treatment [11].

Although rare, *Brucella* endocarditis is responsible for approximately 80% of brucellosis-related mortality [19]. In our case, the outcome was favorable after six months of follow-up thanks to a combination of medical and surgical treatments [3]. The third observation illustrates systemic brucellosis revealed by hepatic decompensation in a cirrhotic patient. Cirrhosis is a state of immunosuppression that exposes patients to serious and sometimes atypical infections. For these patients, it is crucial to consider not only common infections but also endemic local infections, such as brucellosis in Tunisia. The clinical presentation can be misleading. In our case, for example, encephalopathy masked the initial infectious nature of the disease. Targeted questioning and identification of risk factors, particularly raw milk consumption, were decisive in guiding the diagnosis. This observation underscores the importance of including endemic pathogens in the diagnostic approach for immunocompromised patients to prevent delayed diagnoses and serious complications.

A common feature of our three cases is the mode of contamination, which is linked to consuming unpasteurized raw milk. This finding underscores the importance of preventive measures at the human-animal interface. Within the framework of the One Health approach, which recognizes the interdependence of human, animal, and environmental health. These observations underscore the importance of preventive measures at both human and animal levels [1]. Therefore, it is essential to support veterinary and livestock services by allocating the necessary human, financial, and logistical resources to better control the animal aspect of the disease, particularly through herd monitoring, vaccination, and pasteurization of dairy products.

It should be noted that transmission can occur during animal care or slaughter, exposing individuals to various clinical forms of brucellosis, some of which can be severe, such as systemic or osteoarticular forms [9].

Raising public awareness of these risks and implementing strict veterinary control measures are major levers for reducing the incidence of human brucellosis in endemic areas. This strategy emphasizes promoting the consumption of pasteurized dairy products, improving herd health monitoring, and enforcing strict hygiene practices during breeding and slaughter.

## Conclusion

Human brucellosis can mimic many infectious or inflammatory diseases, which makes diagnosis difficult in some cases. In endemic areas such as Tunisia, practitioners must systematically consider and investigate it, particularly in at-risk patients (those living in rural areas, consuming unpasteurized dairy products, or working in specific occupations). High clinical suspicion combined with close collaboration between clinicians and microbiologists is essential for rapid diagnosis and effective management.

## Informed consent

We obtained verbal consent from the patients.

## Funding

This work did not receive any funding.

## Author’ contributions

Manel Ennaceur: Design of the case report, diagnostic management, writing, and manuscript revision.

Khaoula Souli: diagnostic management and writing.

Sonia Chouaieb: manuscript revision and validation.

## Conflicts of interest

The authors declare no conflicts of interest.
